# Supplementary material for: Finger-Based Numerical Training Increases Sensorimotor Activation for Arithmetic in Children—An fNIRS Study
Source: Brain Sci. 2022 May 12;12(5):637. doi: 10.3390/brainsci12050637 (PMC9139726; doi:10.3390/brainsci12050637)
Supplement: Supplementary file 1 [file brainsci-12-00637-s001.zip › brainsci-1608094-supplementary.pdf]

SUPPLEMENTARY MATERIAL ON THE NUMBER PARTNER TASK

Title: Finger-based numerical training increases sensorimotor activation for arithmetic in children – An fNIRS study

Authors: Christina Artemenko, Silke Maria Wortha, Thomas Dresler, Mirjam Frey, Roberta Barrocas, Hans-Christoph Nuerk, Korbinian Moeller

Introduction

Finger calculation is most likely performed through ordinal and/or cardinal strategies. Respectively, these correspond to counting on each finger individually or simultaneously depicting numbers as finger patterns. By comparing the number of raised and folded fingers on a finger pattern, children may learn how different operands relate to the wholeness of the problem's cardinal structure (Gattegno, 1974). In fact, many kindergarten children use so-called part-whole finger-based strategies for solving basic arithmetic tasks (Björklund et al., 2019). Moreover, kindergarten children trained in part-whole finger-based strategies saw significant improvements in their arithmetic learning compared to a passive control group (Kullberg et al., 2019). Thus, part-whole relations in finger patterns are related to arithmetic skills.

In the present study, we further explored whether trained children differed in their sensorimotor and IPS activation for in part-whole relations from control children who did not complete the finger-based numerical training by focusing on number partner (i.e., which numbers add up to 10).

Methods

Experimental task. In the number partner task, number partners were defined as number pairs adding up to 10 when combined (e.g., 3 and 7). Items consisted of single-digit numbers with their respective number partner, both ranging from 1 to 9. In each trial, a single-digit number was presented and participants had to indicate the corresponding number partner. The task consisted of three conditions: (1) In the verbal condition, a number was presented in Arabic notation on the screen and participants responded orally by saying "is [number partner]". (2) In the finger condition, a number was presented in Arabic notation on the screen and participants responded by displaying the finger pattern corresponding to the number partner with their fingers and simultaneously pressing large response keys with their whole hands (with the ball of the thumb). (3) In the motor control condition for the finger condition, hand pictures with one marked finger (except for the thumb) were presented on the screen and participants were instructed to touch their corresponding finger with the thumb of the same hand and simultaneously pressing one of the large response keys with their whole hand (with the ball of the thumb). In a block design, each block lasted for 36 s with an inter-block interval of 20 s. Within each block, each of the 9 stimuli per condition was presented in random order for 3.5 s, followed by an inter-stimulus-interval of 0.5 s. There were 6 blocks in total which followed the order of 2 blocks of the finger condition, 2 blocks of the verbal condition, and 2 blocks of the motor condition.

Data analysis. In the number partner task, participants were excluded from behavioral data analysis due to missing data in all conditions ($n = 2$), and from RT analysis only due to missing or less than 20% valid RT data in the verbal condition ($n = 1$), in the finger condition ($n = 12$), or in the motor condition ($n = 1$). Note that we had problems with the response format (finger movements and simultaneous button press within a time limit) and documentation in this task so that the correctness of the behavioral data cannot be assured. Therefore, we report this data only in the Supplementary Material. The number partner task further followed a block

design so that incorrectly solved trials were not excluded from the fNIRS data analysis. During fNIRS data preprocessing, 13.79% of the fNIRS channels were interpolated in the number partner task. The block fNIRS data of the partner number task was analyzed by block averages considering the mean amplitude during the block between 3-36 s and a preceding baseline of 0.5 s for each channel, participant, and condition.

The behavioral data (RT and accuracy) of the number partner task was analyzed by independent sample *t*-tests comparing the groups (trained vs. control children) separately for the verbal, finger and motor conditions. For the fNIRS data, ANOVAs discerning the factors group (trained vs. control children) and hemisphere (left vs. right) were conducted separately for the verbal, finger and motor conditions for each ROI (sensorimotor cortex and IPS). Post-hoc *t*-tests were added when necessary.

Results

Behavioral data. The RT analysis revealed no significant group differences in the verbal, $t(39) = 0.811$, $p = .422$, finger, $t(15.81) = 1.125$, $p = .277$, or motor condition, $t(39) = 0.811$, $p = .422$. The analysis of accuracy also indicated no significant group differences in the verbal, $t(40) = -1.486$, $p = .145$, finger, $t(40) = -1.027$, $p = .311$, or motor condition, $t(40) = -1.467$, $p = .453$. Note that for the finger condition a Welch *t*-test was conducted for RT, since the Levene's test indicated unequal variances of the groups ($p = .001$).

fNIRS data. For the sensorimotor cortex, a significant main effect for group, $F(1, 42) = 7.27$, $p = .010$, $\eta_p^2 = 0.148$, was qualified by a significant interaction of group and hemisphere, $F(1, 42) = 4.29$, $p = .044$, $\eta_p^2 = 0.093$. Simple effects analyses indicated that trained children showed significantly higher activation than control children in the left sensorimotor cortex, $t(42) = 3.16$, $p = .003$, $d = 0.951$, but not in the right sensorimotor cortex, $t(42) = 1.28$, $p = .209$ (see Figure S1A). The main effect of hemisphere was not significant in the verbal condition, $F(1, 42) = 2.37$, $p = .131$, $\eta_p^2 = 0.054$.

In the finger condition, neither the main effects for group, $F(1, 42) = 0.898$, $p = .349$, $\eta_p^2 = 0.021$, or hemisphere, $F(1, 42) = 0.199$, $p = .658$, $\eta_p^2 = 0.005$, nor their interaction was significant, $F(1, 42) = 1.52$, $p = .255$, $\eta_p^2 = 0.035$. In the motor condition, the main effect of group was only marginally significant, $F(1, 42) = 4.07$, $p = .050$, $\eta_p^2 = 0.088$, indicating sensorimotor activation might be higher for trained children as compared to control children. No significant effects were found for hemisphere, $F(1, 42) = 0.012$, $p = .913$, $\eta_p^2 < 0.001$, or the interaction, $F(1, 42) = 0.033$, $p = .858$, $\eta_p^2 < 0.001$.

For the *IPS*, a significant interaction of group and hemisphere was found in the finger condition, $F(1, 42) = 6.59$, $p = .014$, $\eta_p^2 = 0.136$, indicating that lateralization of activation was more pronounced in trained as compared to untrained children. Simple effect indicated that trained children showed a larger activation in the right IPS than left IPS, $t(21) = 3.05$, $p = .006$, $d = 0.650$, while no significant lateralization effects were found for control children, $t(21) = -0.851$, $p = .405$ (see Figure S1B). No significant main effects of group, $F(1, 42) = 0.007$, $p = .933$, $\eta_p^2 < 0.001$, and hemisphere, $F(1, 42) = 1.53$, $p = .223$, $\eta_p^2 = 0.035$, were observed in the finger condition.

In the verbal condition, no significant main effects of group, $F(1, 42) = 0.417$, $p = .522$, $\eta_p^2 = 0.010$, and hemisphere, $F(1, 42) = 0.032$, $p = .860$, $\eta_p^2 < 0.001$, were observed. Additionally, the interaction was not significant, $F(1, 42) = 1.03$, $p = .316$, $\eta_p^2 = 0.024$. Similarly, in the motor condition, no significant main effects for group, $F(1, 42) = 0.026$, $p = .872$, $\eta_p^2 < 0.001$, or hemisphere, $F(1, 42) = 2.53$, $p = .119$, $\eta_p^2 = 0.057$, were observed. Additionally, the interaction was not significant, $F(1, 42) < 0.001$, $p = .985$, $\eta_p^2 < 0.001$.

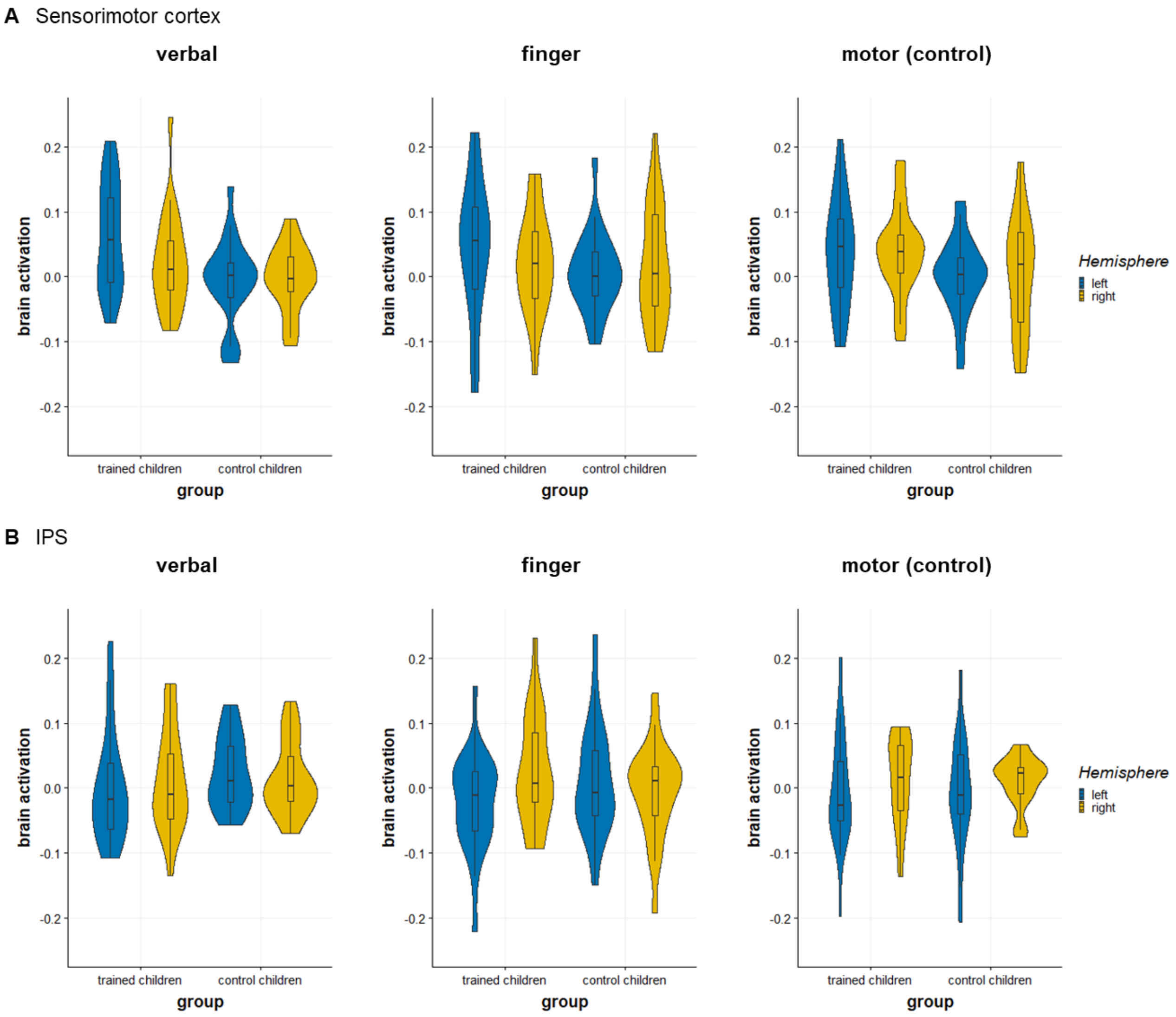

Figure S1. Neural activation of the number partner task separated for in the verbal, finger and motor control condition. (A) Sensorimotor cortex: In the verbal condition, trained children showed higher activation in the left sensorimotor cortex than control children. No significant effects were observed in the finger and motor control conditions. (B) IPS: In the finger condition, lateralization in the IPS were observed only for trained children. No significant effects were observed in the verbal and motor control conditions. Violin plots display the kernel distribution of the neural activation within each ROI; box plots include the interquartile range (IQR = 25–75%) with the horizontal line representing the median.

Discussion

For part-whole relations, observed activation differences due to the finger-based intervention were in line with the results for single-digit arithmetic. Larger activation of the left sensorimotor cortex was found in trained as compared to control children in the absence of overt finger movements. Activation differences in the IPS between trained and control children were less conclusive. Overall, converging evidence for finger-based numerical representations was provided by both tasks, however, the findings presented here need to be interpreted with caution due to the untrustworthy behavioral basis.

Corroborating the findings for mental arithmetic, more pronounced activation in the left sensorimotor cortex was observed for trained as compared to control children while enunciating partner numbers. This

finding might be explained by the involvement of the dominant hand (i.e., right hand in right-handers) in all single-digit numbers, while the other hand is needed only in showing single-digit numbers larger than 5. When children were trained in using their fingers for part-whole relations, left sensorimotor activation subserving finger movements of the right hand might reflect the larger involvement of the dominant hand in representing single-digit numbers (cf. Tschentscher et al., 2012). In the absence of overt finger movements, the more pronounced sensorimotor activation for trained children might reflect traces of the finger-based intervention also for part-whole relations up to 10.

The number partner task with fingers elicited more activation in the right than left IPS in trained children (see also Kaufmann et al., 2008). This lateralization of IPS activation might reflect facilitated number magnitude processing in the IPS due to the finger-based training, in agreement with the results of the addition task. However, it might also be explained by the age difference between the groups tested in the present study and thus reflect an age-related increase in left-hemispheric IPS activation during arithmetic development (e.g., Bugden et al., 2012). Therefore, the interpretation of training-induced changes in IPS activation is confounded by developmental changes and future research needs to disentangle these effects.

References

Björklund, C., Kullberg, A., & Kempe, U. R. (2019). Structuring versus counting: critical ways of using fingers in subtraction. *ZDM Mathematics Education*, *51*, 13–24. doi:10.1007/s11858-018-0962-0

Bugden, S., & Ansari, D. (2011). Individual differences in children's mathematical competence are related to the intentional but not automatic processing of Arabic numerals. *Cognition*, *118*, 32–44. doi:10.1016/j.dcn.2012.04.001

Gattegno, C. (1974). *The Common Sense of Teaching Mathematics*. New York: Educational Solutions.

Kaufmann, L., Vogel, S. E., Wood, G., Kremser, C., Schocke, M., Zimmerhackl, L.-B., & Koten, J. W. (2008). A developmental fMRI study of nonsymbolic numerical and spatial processing. *Cortex*, *44*(4), 376–385. doi:10.1016/j.cortex.2007.08.003

Kullberg, A., Björklund, C., Brkovic, I., & Kempe, U. R. (2020). Effects of learning addition and subtraction in preschool by making the first ten numbers and their relations visible with finger patterns. *Educational Studies in Mathematics*, *103*, 157–172. doi:10.1007/s10649-019-09927-1

Tschentscher, N., Hauk, O., Fischer, M. H., & Pulvermüller, F. (2012). You can count on the motor cortex: finger counting habits modulate motor cortex activation evoked by numbers. *NeuroImage*, *59*(4), 3139–3148. doi:10.1016/j.neuroimage.2011.11.037
